# Intrinsic Immune Mechanisms Restricting Human Cytomegalovirus Replication

**DOI:** 10.3390/v13020179

**Published:** 2021-01-26

**Authors:** Eva-Maria Schilling, Myriam Scherer, Thomas Stamminger

**Affiliations:** Institute of Virology, Ulm University, 89081 Ulm, Germany; eva-maria.schilling@uni-ulm.de (E.-M.S.); myriam.scherer@uni-ulm.de (M.S.)

**Keywords:** cytomegalovirus, restriction factor, intrinsic immunity

## Abstract

Cellular restriction factors (RFs) act as important constitutive innate immune barriers against viruses. In 2006, the promyelocytic leukemia protein was described as the first RF against human cytomegalovirus (HCMV) infection which is antagonized by the viral immediate early protein IE1. Since then, at least 15 additional RFs against HCMV have been identified, including the chromatin regulatory protein SPOC1, the cytidine deaminase APOBEC3A and the dNTP triphosphohydrolase SAMHD1. These RFs affect distinct steps of the viral replication cycle such as viral entry, gene expression, the synthesis of progeny DNA or egress. This review summarizes our current knowledge on intrinsic immune mechanisms restricting HCMV replication as well as on the viral strategies to counteract the inhibitory effects of RFs. Detailed knowledge on the interplay between host RFs and antagonizing viral factors will be fundamental to develop new approaches to combat HCMV infection.

## 1. Introduction

Host organisms have evolved several layers of interconnected mechanisms to efficiently counteract viral infections. Conventional innate and adaptive immune mechanisms effectively target virus infections, however, both need to be evoked through diverse inducible pathways which delays the response [1]. In contrast, constitutive innate immune mechanisms have the advantage of an immediate host defense against invading pathogens. A large number of constitutive mechanisms of innate immunity have been described including chemical barriers, antimicrobial peptides, basal autophagy or proteasomal degradation [2]. One drawback of these mechanisms is a lack of specificity. Recent research emphasizes the importance of a subgroup of constitutive innate immune mechanisms mediated by host restriction factors (RFs). RFs, alternatively called cell intrinsic immune factors, are characterized by several hallmarks: RFs (i) are dominantly and autonomously acting factors which form a front-line defense against viral infections, (ii) are constitutively expressed in specific cells but may undergo profound upregulation by interferons, (iii) employ unique and virus-specific mechanisms to interfere with viral replication, (iv) exhibit high interspecies diversity due to co-evolution of hosts with different viruses and (v) are often antagonized by viral proteins or virus-specific adaptation mechanisms [1,3]. 

Intrinsic immunity has been extensively studied in the past decades, which led to the discovery of several different host cell effector proteins involved in the intrinsic defense against various viruses. For HIV-1 (human immunodeficiency virus type 1) the most prominent RFs are the E3 ubiquitin ligase TRIM5α and the cytidine deaminase APOBEC3G, as well as SAMHD1 and tetherin, which efficiently restrict distinct steps during HIV-1’s life cycle [4,5,6,7,8,9]. Some of these RFs as exemplified by TRIM5α are well known to act as an efficient barrier for cross-species transmission [10]. Other examples of RFs are the MxA protein and the IFIT (interferon-induced proteins with tetratricopeptide repeats) and IFITM (interferon-induced transmembrane) family members, which were identified as important players in the defense against influenza virus infection [9,11,12,13,14].

HCMV (human cytomegalovirus), a ubiquitous β-herpesvirus, can cause severe disease in individuals with immature or compromised immune systems and in congenitally infected children. The outcome of HCMV infection is determined by a number of factors, some related to the virus, others to the host, which include immunogenetic factors [15]. Its double-stranded DNA genome contains >200 protein-coding genes and a large number of HCMV gene products have been found to function in immune evasion including the antagonization of host RFs [16,17,18]. In 2006, the PML (promyelocytic leukemia) protein, the defining constituent of the nuclear domain 10 (ND10), was described as the first RF against HCMV [19,20]. Since then, numerous additional host RFs have been identified acting at distinct steps of the viral replication cycle. This short review summarizes our current knowledge on intrinsic immunity defending against HCMV infections. In addition, we also provide a description of the viral mechanisms used to modulate and counteract host RFs. 

## 2. Galectin-9 Restricts the Entry of HCMV into Host Cells

**Figure 1:**
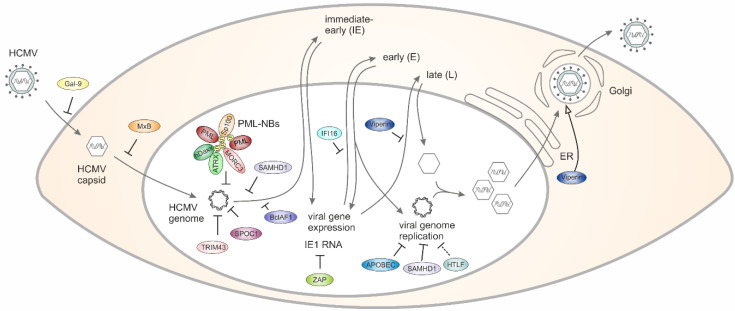
Schematic outline of the strategies used by host RFs (restriction factors) to counteract HCMV (human cytomegalovirus) replication.

Galectin-9 (Gal-9), a member of a family of secreted glycan-binding proteins with a conserved carbohydrate recognition domain (CRD), is known for its pleiotropic immune-regulatory properties [21]. Galectins are able to bind glycan structures on the surface of cells and this can modulate a variety of diverse functions including cell–cell interactions, cell–matrix adhesion, or transmembrane signaling. First evidence for a role of Gal-9 during HCMV infection was obtained by monitoring blood samples of hematopoietic stem cell recipients for Gal-9 mRNA levels. This revealed significantly increased expression of Gal-9 mRNA in patients with HCMV reactivation [22]. Results obtained by in vitro infection of primary human fibroblasts demonstrated that a soluble factor mediates the upregulation of Gal-9 and finally interferon (IFN)-β was identified as a mediator of Gal-9 induction during HCMV infection [22]. In an attempt to evaluate the impact of Gal-9 on HCMV replication, primary human fibroblasts as well as retinal pigment epithelial cells were infected in the presence of increasing concentrations of Gal-9. This revealed a dose-dependent downregulation of HCMV infection which was specific for Gal-9 and could be observed in a cell-type and HCMV-strain-independent manner [23]. Mechanistically, downregulation required Gal-9 lectin binding and was mediated primarily via an interaction with virions. Furthermore, the authors provided evidence for HCMV entry inhibition by Gal-9 occurring at the level of virus–cell fusion [23]. Importantly, HCMV-dependent upregulation of soluble Gal-9 could be demonstrated in hematopoietic stem cell transplant patients suggesting that Gal-9 may function as an important antiviral defense effector molecule in vivo [23]. In summary, Gal-9 constitutes the first cellular RF against HCMV which operates via entry inhibition (Figure 1, Table 1).

## 3. MxB Interferes with the Nuclear Delivery of HCMV Genomes

Mx proteins are IFN-induced dynamin-like GTPases that consist of a globular GTPase domain connected to an extended stalk. While MxA is well known for its restriction of a large panel of positive- and negative-strand RNA and certain DNA viruses but, notably, not herpesviruses or HIV-1 (reviewed in [24]), initial studies suggested that MxB lacks antiviral activity. A first report connecting this GTPase with antiviral restriction of herpesviruses identified MxB in a screen for IFN-induced genes conferring resistance to murine gammaherpesvirus 68 (MHV68) [25]. In 2018, Crameri et al. provided direct evidence that ectopic MxB expression restricts lytic replication of HSV (herpes simplex virus)-1 and HSV-2 as well as latent infection of KSHV [26]. Further experiments revealed that MxB interferes with the delivery of herpesviral genomes into the nucleus, a step that requires the GTPase function of MxB [26]. Shortly thereafter, two further studies confirmed and even extended the anti-herpesviral activity of MxB to HCMV and MCMV [27,28]. Moreover, it could be demonstrated that, in addition to the GTPase domain, the stalk region is required for MxB’s anti-herpesviral activity presumably by mediating dimerization or even oligomerization via self-assembly [27]. Altogether, all three studies published in 2018 collectively identified MxB as a novel pan-herpesvirus RF that acts as a postentry inhibitor interfering with the delivery of herpesviral genomes into the host cell nucleus (see Figure 1) [29].

## 4. PML Nuclear Bodies Associate with Parental HCMV Genomes to Induce Epigenetic Silencing

PML protein, a member of the tripartite motif (TRIM) protein family, represents the key component of subnuclear structures known as PML nuclear bodies (PML-NBs) or nuclear domain 10 (ND10). Since PML-NBs consist of numerous permanently or transiently associated proteins, they have been implicated in the regulation of diverse cellular processes including cell cycle, apoptosis, senescence, stress and DNA damage responses [30]. The role of PML-NBs in intrinsic immunity was discovered many years ago and has been extensively characterized in the context of herpesviral infections. As shown for other herpesviruses, HCMV genomes become associated with PML-NBs after entering the cell nucleus (Figure 1) [31,32]. This association results in epigenetic silencing of HCMV genomes and, thus, blocks the initiation of lytic replication (Table 1). Employment of knockdown or overexpression techniques in numerous studies has identified several PML-NB components, including PML, Sp100, hDaxx and ATRX as RFs that contribute to the silencing of HCMV DNA by recruiting chromatin-modifying enzymes (reviewed in [20,33]). 

During lytic infection, HCMV expresses two regulatory proteins that act in a sequential manner to efficiently antagonize PML-NB-based repression. Initially, the tegument-delivered protein pp71 is imported into the nucleus where it induces dissociation of ATRX from PML-NBs [34]. This is followed by pp71-induced degradation of hDaxx, which occurs in a ubiquitin-independent but proteasome-dependent manner and facilitates initiation of viral IE gene expression [35,36]. Subsequently, the immediate early protein IE1 is expressed and induces a complete dispersal of PML-NBs [37]. Mechanistic studies have revealed that IE1 directly interacts with PML through its α-helical core domain and blocks the SUMOylation of PML, which is essential for PML-NB integrity [38,39]. More recently, a novel antagonist of PML-NBs has been identified, which is expressed during HCMV latency [40]. The latency-associated gene product LUNA, which shows deSUMOylase activity, was reported to induce PML deSUMOylation and dispersal of ND10 bodies, thereby promoting HCMV reactivation in response to external stimuli.

Of note, the PML protein emerges as a mediator of both intrinsic and innate immune defenses. In particular, PML was identified as a positive regulator of the IFN pathway, which can enhance the expression of IFN-β and, additionally, is required for an efficient transcription of type I and type II IFN-stimulated genes (ISGs) [41,42,43,44]. Consequently, viral proteins that modulate PML-NBs may not only antagonize intrinsic but also innate immune responses, which has already been demonstrated for IE1 of HCMV [43,44]. Controversial results, however, were provided by a recent study characterizing a recombinant HCMV that expresses a PML-binding deficient mutant of IE1 [45]. Since lower instead of higher cytokine and ISG expression was observed upon infection with the recombinant virus, the authors speculated that disruption of PML bodies may be linked to immune activation. Thus, further experimentation will be required to detect possible virus strain- and cell-type-specific differences in innate immune regulation and to define the exact role of PML during HCMV infection.

## 5. MORC3 Affects the Recruitment of PML-NB Components to Viral DNA

In 2016, the protein Microorchidia 3 (MORC3) was described as a novel RF against HSV-1 and HCMV [46]. MORC3 is a SUMOylated nuclear matrix protein with ATPase activity that has been reported to localize to PML-NBs via a SUMO-SIM mediated interaction with PMLI in uninfected cells [47]. Upon infection with HSV-1, MORC3 was found to be recruited to sites associated with HSV-1 genomes [46]. Viral plaque assays revealed antiviral activity, albeit only against ICP0-null mutant HSV-1 since restriction by MORC3 is efficiently antagonized by an ICP0-mediated degradation in a manner that depends on its RING finger domain [46,48]. Additionally, MORC3’s antiviral role extends to HCMV infection as its plaque-forming efficiency increased in MORC3-depleted cells [46]. For HCMV, data on a viral protein antagonizing MORC3 are currently not available. However, a proteomic screen to quantify protein degradation during the early phase of HCMV infection identified MORC3 to be degraded in a proteasomal manner, suggesting the activity of a viral countermeasure [49]. Depletion experiments revealed that MORC3 is required for efficient recruitment of PML, Sp100, hDaxx and γH2AX to viral DNA [46]. Since a recent publication suggested that MORC3 is capable of forming liquid-like nuclear condensates through phase separation, depending on its ATPase activity, one may speculate that MORC3 facilitates the formation of local nuclear structures fueling the epigenetic silencing of herpesvirus genomes [50]. 

## 6. TRIM43 Represses Active Viral Chromatin States via Ubiquitination and Degradation of Pericentrin

Besides PML, another TRIM protein emerges as a potent, herpesvirus-specific antiviral factor: by performing an RNAi screen, Full et al. identified 15 TRIM proteins that suppress KSHV reactivation. Among them, the centrosomal protein TRIM43 was distinguished by its role as RF for a broad range of herpesviruses, including HCMV [51]. TRIM43 expression is induced by herpesvirus infection, which displays a hallmark of antiviral factors. In contrast to many other RFs, however, this upregulation does not depend on IFN signaling but is part of a germline-specific transcriptional program mediated by the transcription factor DUX4. Mechanistically, the authors provided evidence that TRIM43 ubiquitinates the centrosomal protein pericentrin by utilizing its RING E3 ligase activity, thereby targeting it for proteasomal degradation [51]. This subsequently induces alterations of the nuclear lamina and leads to a repression of active viral chromatin states. Since an increase of HCMV IE gene expression was observed upon TRIM43 knockdown, it can be concluded that one of the first steps in the HCMV replication cycle is affected via the TRIM43–pericentrin–lamin axis. 

## 7. SPOC1 Associates with the HCMV Major Immediate Early Promoter to Induce Chromatin Compaction

SPOC1 (survival-time associated PHD protein in ovarian cancer 1), also known as PHF13 (PHD finger 13), was first described in 2005 as a novel cellular protein with a single plant homeodomain (PHD) showing high expression in ovarian carcinoma patients which correlated with poor prognosis [52]. Subsequent studies revealed that this protein acts as a regulator of the DNA damage response and of chromatin structure via binding to H3K4me2/3-containing chromatin. This recruits corepressors such as KAP1 and histone methyltransferase SETDB1 to promote chromatin compaction [53,54]. Apart from its cellular regulatory functions, studies on human adenovirus type 5 (HAdV5) provided first evidence that SPOC1 contributes to the intrinsic defense against viral infections [55]. Schreiner and colleagues observed that HAdV5 gene expression was diminished upon overexpression of SPOC1, suggesting that repression takes place at the transcriptional level. Furthermore, human adenoviruses antagonize this repression by inducing the proteasomal degradation of SPOC1 early after infection [55]. For HCMV, a highly specific association of SPOC1 with the major immediate early promoter (MIEP) could be detected by chromatin immunoprecipitation sequencing, suggesting that SPOC1 represses HCMV replication by MIEP binding and recruitment of heterochromatin-building factors [56]. Consistently, SPOC1 overexpression severely impaired HCMV replication while SPOC1 depleted cells displayed an augmented initiation of viral immediate early gene expression. In contrast to adenovirus infection, SPOC1 was not degraded during infection but a transient upregulation of SPOC1 during the early phase of HCMV infection was observed [56]. However, since only high SPOC1 levels at the start of infection mediate efficient repression, HCMV may have evolved alternative antagonistic mechanisms operating during later phases of viral replication that require further investigation. Since SPOC1 expression levels exhibit a considerable variation between cell types, one may assume that this protein endows specific tissues of an organism with an additional RF against HCMV acting via chromatin compaction.

## 8. IFI16 Targets Incoming HCMV DNA to Regulate Viral Promoter Activities 

IFI16 is an IFN-inducible and predominantly nuclear protein that, as a member of the PYHIN protein family, contains an N-terminal pyrin domain (PYD) and two partially conserved 200-residue domains (HIN domains) in the C-terminus. While the α-helical PYD promotes homotypic protein interactions with other PYD-containing proteins, the HIN domains allow an interaction with both dsDNA and ssDNA in a sequence-independent manner [57,58,59]. During HCMV infection, the viral dsDNA genome is recognized by IFI16 resulting in a colocalization inside the cell nucleus [60]. Since live-cell studies revealed a re-localization of IFI16 to nuclear peripheral foci within the first hours of HCMV infection, it can be assumed that IFI16 rapidly targets incoming HCMV DNA. The authors furthermore reported that IFI16 oligomerizes at sites of herpesviral DNA deposition in order to regulate viral gene expression and limit viral replicative capacity [61]. Whereas the recruitment of IFI16 appears to activate the major immediate early promoter (MIEP) immediately upon HCMV infection, IFI16 displays a restrictive activity at later times as it suppresses expression of the HCMV DNA polymerase (UL54) and its processivity factor (UL44), which are required for viral DNA synthesis [62,63,64]. The molecular mechanism of the latter, antiviral activity of IFI16 is based on its ability to bind and block Sp1-like transcription factors on viral promoters [64]. Notably, the interaction of HCMV tegument protein pp65 (pUL83) with IFI16 was shown to be required for both the positive and negative regulation of HCMV promoter activity [62,63]. At late stages of infection, pp65 additionally modulates IFI16 function as it induces a re-localization into the cytoplasm thereby antagonizing the antiviral activity of IFI16 inside the nucleus [62]. As a second viral regulator, the HCMV phosphoprotein UL97 has been demonstrated to bind and phosphorylate IFI16, which promotes the nucleo-cytoplasmic re-localization of this protein and finally results in translocation to the virus assembly complex and incorporation into newly formed virions [60].

Besides its role as HCMV RF, IFI16 participates in the innate immune defense by acting as a pattern recognition receptor: IFI16 binds nuclear HCMV dsDNA and triggers expression of antiviral cytokines via the STING/TBK1/IRF signaling pathway [65]. Again, this response is blocked by pp65, which sequesters the IFI16 PYD and blocks nuclear IFI16 oligomerization and subsequent immune signaling [65]. Thus, the interaction with pp65 appears critical for the outcome of HCMV infection as it modulates the intrinsic and innate immune activities of IFI16. 

## 9. BclAF1 Restricts Herpesviral Replication via Regulation of the Type I Interferon Response

BclAF1 (Bcl-2-associated transcription factor 1), alternatively termed Btf (Bcl-2 associated transcription factor) was first identified by yeast two-hybrid screening as a binding partner of the adenovirus E1B 19 k protein [66]. Multiple cellular functions have been described for this nuclear protein including induction of apoptosis, repression of transcription or regulation of alternative splicing [67]. Concerning its role during HCMV infection, Lee and colleagues depleted BclAF1 by transfection of siRNAs and observed increased IE1 protein expression without affecting pp71 levels suggesting that BclAF1 may repress viral IE gene expression [68]. Furthermore, they identified two distinct viral mechanisms to antagonize BclAF1: firstly, BclAF1 was shown to undergo proteasomal degradation immediately after infection. This was dependent on virion proteins including pp71 [68]. Secondly, BclAF1 expression was found to be downregulated by the HCMV microRNA miR-UL112-1 which reduces BclAF1 protein levels. Consistently, infection of cells expressing a BclAF1 transcript with a 3’UTR lacking the miR-UL112-1 binding site prevented HCMV spread, further suggesting that BclAF1 acts as physiologically relevant RF that needs to be neutralized by virus-specific mechanisms [68]. Recent studies indicate that downregulation of BclAF1 is not specific for HCMV but also occurs during infection with pseudorabies virus and HSV-1 [69]. Importantly, this study revealed that BclAF1 acts as an important host factor for IFNα-induced ISG expression via facilitating the phosphorylation of STAT1/STAT2 and via a direct interaction with STAT2 at ISG promoters [69]. Thus, BclAF1 may primarily restrict herpesvirus replication in an indirect manner through regulation of the IFN-mediated antiviral response. 

## 10. HCMV Evades ZAP Detection by Suppressing CpG Dinucleotides in the Major Immediate Early 1 RNA

To avoid recognition as foreign nucleic acids, genomes of mammalian RNA and small DNA viruses mimic the composition of their host genomes by significantly suppressing CpG dinucleotide frequencies, whose artificial increase, however, results in considerable attenuation of virus replication [70,71,72,73]. Recently, the IFN-inducible Zinc finger antiviral protein (ZAP) was identified as host factor responsible for sensing CpG in viral RNA, through direct binding and possibly downstream targeting for degradation [74]. A recent arrayed ISG expression screening performed by Lin and colleagues identified ZAP to restrict HCMV in a manner that is independent of IRF3. Overexpression and knockdown experiments displayed decreased or increased virus replication, respectively, further confirming ZAP as a RF against HCMV [75]. For herpesviruses, the pattern of CpG dinucleotide frequencies is distinct: while the majority of alpha-herpesviruses demonstrate little or no CpG suppression, gamma-herpesviruses exhibit substantial suppression across the genome. Interestingly, beta-herpesviruses display the most striking pattern, with suppression of CpG dinucleotides confined to gene regions expressed with immediate early kinetics [76]. Consistently, scanning analysis demonstrates that the IE1 gene is the only region of the HCMV genome that is suppressed for CpG content [76]. In accordance, Lin et al. demonstrated that HCMV transcripts with high CpG content are specifically targeted by ZAP, while the CpG-suppressed IE1 transcript remains unaffected [75]. However, artificially increasing the IE1 CpG content by introducing mutations into the IE1 coding region renders IE1 accessible to ZAP inhibition. Interestingly, subsequent analyses revealed that endogenous ZAP is induced during HCMV infection but its expression is mutually exclusive to acute virus progression. The authors further speculated that higher levels of CpG in viral genes expressed subsequent to IE1 result from the loss of ZAP-mediated pressure in infected cells [75].

## 11. HTLF is Degraded during HCMV Infection to Avoid Post-Replicative DNA Repair

Helicase-like transcription factor (HTLF), a member of the switch/sucrose non-fermenting (SWI/SNF) family, was first identified as a DNA binding protein interacting with motifs of the SV40 enhancer and HIV-1 promoter [49]. Subsequent studies associated this cellular factor with the prognosis of a number of cancer types leading to the hypothesis that HTLF may act as a tumor suppressor [77]. Mechanistically, HLTF was shown to function as a ubiquitin ligase for DNA replication processivity factor PCNA to ensure error-free post-replication repair of damaged DNA replication forks [78]. A recently performed high-definition analysis to quantify protein stability during HCMV infection detected that HTLF is targeted for degradation at very early times of the replicative cycle starting from 4 h [49]. The authors postulated that HLTF might undergo early degradation in order to annihilate antiviral restriction instituted by this protein. In order to demonstrate antiviral restriction by HLTF, shRNA-mediated knockdown was performed revealing enhanced HCMV infection in HLTF-depleted cells under conditions of low multiplicity of infection. Furthermore, it could be shown that the viral protein UL145 recruits the cullin E3 ligase complex to target HTLF for proteasomal degradation [49]. Of note, this resembles the Vpr protein of HIV-1 which reprograms the CRL4^DCAF1^ E3 ubiquitin ligase to degrade HLTF [79]. Interestingly, a recent publication demonstrated restriction of HIV-1 replication in T-cells by HLTF which was dependent on a functional HIRAN domain suggesting that sensing and processing of fork-like branched DNA structures by HLTF interfere with ordered progression of plus strand synthesis [80]. Although not formally proven, one may speculate that a similar mechanism of restriction by the HLTF DNA helicase may also apply to HCMV which replicates through a double-stranded DNA intermediate.

## 12. APOBEC3 Proteins Induce Cytidine-Deaminase Mediated Hypermutation of the HCMV Genome in a Cell-Type Specific Manner

The apolipoprotein B editing enzyme catalytic subunit 3 (APOBEC3) family of proteins is known for more than a decade for its strong antiviral activity against HIV-1 [3]. APOBEC3G constitutes the prototype antiretroviral cytidine deaminase which acts during reverse transcription to preferentially deaminate the third cytosine of the single-stranded DNA sequence 5′-CCCA-3′ [81]. This disrupts the coding potential of the viral genome, generally rendering it replication defective [3]. Other APOBEC3 proteins can also restrict lentiviruses via their hypermutation activity but differ in their domain organization, sequence preferences and their propensity to utilize deaminase-independent mechanisms [82]. In addition to retroviruses, the replication of a number of DNA viruses including hepatitis B virus and human papillomaviruses were reported to undergo hypermutation-induced inhibition by APOBEC3 proteins [82]. Only recently, APOBEC3 proteins were also implicated in the restriction of HCMV infection: using a unique ex vivo organ culture model of native human decidual tissue, Weisblum and colleagues observed that the APOBEC3A isoform is profoundly upregulated in HCMV-infected decidual cells. Since this was not the case either in chorionic villi or in HCMV permissive cell lines, upregulation of APOBEC3A may constitute a specific antiviral restriction mechanism of the maternal–fetal interface [83]. Importantly, cytidine deamination editing of the HCMV genome was found to be required for inhibition of HCMV replication both in cell culture models and in vivo during congenital infection, strongly suggesting that APOBEC3A acts as an important anti-HCMV host factor in the maternal–fetal interface [83]. In contrast to HIV-1, which encodes the Vif protein as a well-characterized antagonist of APOBEC3-mediated deamination, no antagonistic proteins are known for HCMV so far [84]. However, a recent study by Pautasso and colleagues, which described an IFN-β mediated induction of APOBEC3G in HCMV infected fibroblasts, suggested that HCMV may have evolved mutational robustness by limiting the presence of APOBEC3G hot spots in essential open reading frames [85]. This may constitute a novel viral strategy to evade the cellular restriction of APOBEC3 proteins. 

## 13. SAMHD1 Restricts HCMV Replication by Limiting NF-kB Activation and Intracellular Deoxynucleoside Triphosphate Pools

The sterile alpha motif (Sam) and histidine-aspartate (HD) domain-containing protein 1 (SAMHD1) is best known and most extensively characterized for its ability to restrict HIV-1 particularly in non-dividing myeloid cells such as macrophages and dendritic cells (DCs) as well as resting CD4^+^ T cells [86,87,88]. In the meantime, further viruses restricted by SAMHD1 have been identified, among them HSV-1, HCMV and EBV [89,90,91,92]. For HSV-1, the authors observed increased viral DNA replication in the absence of SAMHD1 in primary human monocyte-derived macrophages and in differentiated macrophage cell lines, thereby providing a mechanism of restriction that relies on SAMHD1’s dNTP triphosphohydrolase activity leading to depletion of intracellular dNTPs [89,90]. While the addition of exogenous deoxynucleosides partially overcomes the restriction, the absence of viral genes that are involved in dNTP metabolism such as thymidine kinase leads to a more potent suppression by SAMHD1 [89,90]. Interestingly, studies performed by the Weitzman group revealed an additional mechanism of HCMV restriction in permissive fibroblasts and conditionally permissive myeloid cells [93]. They observed that HCMV is restricted by SAMHD1 through inhibition of viral gene expression, which is achieved by inhibiting nuclear factor κB (NF-κB) activation [93]. 

In the last year, mechanisms of SAMHD1 antagonization during herpesvirus infection were identified by several independent groups: conserved herpesvirus protein kinases from all beta- and gamma-herpesviruses (HHV-6/7 U69, HCMV UL97, EBV BGLF4 and KSHV ORF36) were shown to induce a strong phosphorylation of SAMHD1 thereby converting it to its inactive form [91,92]. An additional study utilizing a SAMHD1 knockout mouse model further revealed that MCMV is targeted by SAMHD1 in vitro and that SAMHD1 restricts the replication of MCMV in vivo, thereby underlining the important role of SAMHD1 for CMV restriction [94]. In accordance to findings on HCMV, the authors could demonstrate that MCMV likewise developed countermeasure mechanisms involving the viral kinase M97 [94]. Only recently, the Ahn group provided an additional mechanism of viral countermeasure against SAMHD1-mediated intrinsic defense. They observed that the steady-state SAMHD1 protein level is reduced at late times of infection through a mechanism dependent on Cullin-RING-E3 ligase complexes [95].

## 14. HCMV Redirects the RF Viperin to Enhance Viral Infectivity

Viperin (virus inhibitory protein, endoplasmic reticulum-associated, interferon-inducible), also known as RSAD2 (cig-5), is an IFN-inducible protein that exerts antiviral activity against different viruses. It belongs to the radical S-adenosylmethionine (SAM) superfamily of enzymes [96]. Recent studies demonstrate that viperin catalyzes the transformation of cytidine triphosphate to its analogue 3′deoxy-3′,4′-didehydro-CTP which inhibits the NAD^+^ dependent activity of metabolic enzymes [97]. Initially, it was described as a RF against HCMV, as stable expression of the protein in fibroblasts inhibits productive infection by downregulating the expression of several structural proteins (gB, pp28 and pp65) known to be indispensable for viral assembly and maturation [98]. Interestingly, viperin is not only induced by IFN but also by HCMV infection and by the HCMV envelope protein, glycoprotein B (gB) [98]. Moreover, it has been shown that HCMV infection causes the redistribution of the induced viperin from its normal endoplasmic reticulum association, first to the Golgi apparatus and then to cytoplasmic vacuoles containing gB and pp28, thereby potentially evading the antiviral effects of viperin [98]. Surprisingly, a later study proposed that HCMV even co-opts viperin to enhance its own infectivity [99]. This is achieved by disrupting the metabolism of infected cells: viperin interacts with the HCMV-encoded viral mitochondrion-localized inhibitor of apoptosis (vMIA) causing the re-localization of viperin from the endoplasmic reticulum to the mitochondria [99]. There, viperin interacts with and blocks the function of the mitochondrial trifunctional protein that mediates β-oxidation of fatty acids to generate adenosine triphosphate (ATP) resulting in reduced cellular ATP levels. This leads to actin cytoskeleton disruption and enhancement of infection [99]. Later on, the Creswell group further proved that viperin, presumably as a major effector, regulates cellular lipid metabolism during HCMV infection [100]. They demonstrated that decreased ATP levels activate the enzyme AMP-activated protein kinase, thereby inducing a cascade of events starting with expression of the glucose transporter GLUT4. As a consequence, increased glucose import and activation of glucose-regulated transcription factor ChREBP were observed. This culminated in increased transcription of genes responsible for lipid synthesis, which boosts viral envelopment [100]. 

## 15. Conclusions

During the last few years, several new RFs against HCMV have been identified. The present panel of host intrinsic immune proteins comprises well known players like SAMHD1 or APOBEC3 family members but also factors like HTLF which are so far unique for HCMV. For many RFs, the exact mechanism of restriction has been only partially elucidated. For instance, while Gal-9 restricts HCMV by inhibition of virus–cell fusion, the lectin interacting with Gal-9 on the surface of virions remains to be identified. Similarly, MxB affects the nuclear import of viral DNA but the viral structures or processes targeted by MxB are unknown so far. Another open question is the relative contribution of individual RFs to the overall defense which may vary depending on the infected tissue or organ. For instance, several of the identified RFs (e.g., PML-NBs, SPOC1, TRIM43, BclAF1) converge on chromatin compaction leading to a silencing of gene expression, however, the role of individual silencing mechanisms for the overall antiviral defense remains to be determined. Finally, significant progress has been made concerning the characterization of evasion strategies identifying a number of viral proteins as antagonists of host RFs. However, further research will be necessary to characterize molecular interactions in detail. This will be a prerequisite to develop novel therapeutic agents either boosting host RFs or interfering with viral antagonistic factors.

## Figures and Tables

**Table 1:** Overview of host RFs targeting HCMV.

Restriction Factor		Important Cellular Functions	Major Mechanism of Interference with HCMV Replication	Viral Antagonization—HCMV Effector Protein
Acronym	Detailed Denomination			
Gal-9	Galectin-9	modulation of immune cell responses	entry inhibition	?
MxB	Myxovirus resistance B	integrity of mitochondria	nuclear import of viral DNA	?
PML	Promyelocytic leukemia	tumor suppressor, regulation of stress and DNA damage response	epigenetic silencing	PML deSUMOylation, PML NB-disperal—IE1 (UL123)
Sp100	Speckled protein of 100 kDa	chromatin regulatory protein	epigenetic silencing	PML NB-dispersal, degradation—IE1 (UL123)
Daxx	Death domain associated protein 6	histone H3.3 chaperone (together with ATRX)	epigenetic silencing	proteasomal degradation—pp71 (UL83)
ATRX	Alpha Thalassemia Retardation X-linked	histone H3.3 chaperone (together with Daxx)	epigenetic silencing	displacement from PML NBs —pp71 (UL83)
MORC3	Microorchidia 3	DNA-dependent ATPase, chromatin modulation	recruitment of PML-NB proteins	proteasomal degradation—?
TRIM43	Tripartite Motif Protein 43	ubiquitin E3 ligase, regulation of centrosomal integrity	nuclear lamina alteration to repress active viral chromatin	?
SPOC1	Survival-time associated PHD protein in ovarian cancer-1	H3K4me3 binding, DNA repair, chromatin compaction	silencing of IE transcription	?
IFI16	Interferon gamma inducible protein 16	intracellular DNA sensor, regulation of cellular proliferation and differentiation	regulation of HCMV promoter activities (e.g., downregulation of UL54 expression)	cytoplasmic translocation—pp65 (UL82), UL97
BclAF1	Bcl-2-associated transcription factor 1	regulation of apoptosis, repressor of transcription	regulation of IFN-mediated antiviral response ^1^	proteasomal degradation—pp71 (UL83); microRNA repression—miR-UL112-1
ZAP	Zinc finger antiviral protein	RNA binding, resolution of IFN response, suppression of tumors	degradation of HCMV RNA	CpG suppression in IE1 RNA
HTLF	Helicase-like transcription factor	ubiquitin E3 ligase for PCNA, post- replication repair at DNA forks, tumor suppression	processing of viral replicative intermediates ^1^	proteasomal degradation—UL145
APOBEC3	apolipoprotein B editing enzyme catalytic subunit 3	cytidine deaminase, antiviral defense, cancer mutagenesis	cytidine deaminase mediated hypermutation of HCMV DNA	limited presence of APOBEC3G hotspots in viral genes
SAMHD1	sterile alpha motif and histidine-aspartate domain-containing protein 1	dNTP triphosphohydrolase activity, suppression of autoimmunity, tumor suppressor	depletion of dNTPs, reduced NFkB activation	phosphorylation-mediated inactivation—UL97; proteasomal degradation
Viperin	virus inhibitory protein, endoplasmic reticulum-associated, interferon-inducible	radical SAM enzyme, regulation of cellular metabolism	downregulation of viral late proteins, facilitation of virion morphogenesis	vMIA (UL37x1) co-opts viperin to facilitate viral egress

^1^ proposed mechanism not experimentally proven for HCMV.

## Data Availability

Not applicable.
